# How relative size and abundance structures the relationship between size and individual growth in an ontogenetically piscivorous fish

**DOI:** 10.1002/ece3.3218

**Published:** 2017-07-31

**Authors:** Joshua W. Chamberlin, Brian R. Beckman, Correigh M. Greene, Casimir A. Rice, Jason E. Hall

**Affiliations:** ^1^ NOAA Fisheries Mukilteo Research Station Northwest Fisheries Science Center Mukilteo WA USA; ^2^ NOAA Fisheries Northwest Fisheries Science Center Seattle WA USA

**Keywords:** Chinook salmon, IGF‐1, individual growth, intraguild predation, Pacific herring

## Abstract

While individual growth ultimately reflects the quality and quantity of food resources, intra and interspecific interactions for these resources, as well as individual size, may have dramatic impacts on growth opportunity. Out‐migrating anadromous salmonids make rapid transitions between habitat types resulting in large pulses of individuals into a given location over a short period, which may have significant impact on demand for local resources. We evaluated the spatial and temporal variation in IGF‐1 concentrations (a proxy for growth rate) and the relationship between size and concentration for juvenile Chinook salmon in Puget Sound, WA, USA, as a function of the relative size and abundance of both Chinook salmon and Pacific herring, a species which commonly co‐occurs with salmonids in nearshore marine habitats. The abundance of Chinook salmon and Pacific herring varied substantially among the sub‐basins as function of outmigration timing and spawn timing, respectively, while size varied systematically and consistently for both species. Mean IGF‐1 concentrations were different among sub‐basins, although patterns were not consistent through time. In general, size was positively correlated with IGF‐1 concentration, although the slope of the relationship was considerably higher where Pacific herring were more abundant than Chinook salmon; specifically where smaller individual herring, relative to Chinook salmon, were more abundant. Where Pacific herring were less abundant than Chinook salmon, IGF‐1 concentrations among small and large Chinook salmon were more variable and showed no consistent increase for larger individuals. The noticeable positive effect of relative Pacific herring abundance on the relationship between size and individual growth rates likely represents a shift to predation based on increased IGF‐1 concentrations for individual Chinook salmon that are large enough to incorporate fish into their diet and co‐occur with the highest abundances of Pacific herring.

## 1 INTRODUCTION

In many organisms, individual growth is an outcome of both abiotic (i.e., environmental, physical) and biotic (i.e., food quality and community dynamics) attributes and therefore may differ both spatially and temporally. Growth ultimately reflects the quality and quantity of food resources available to a given individual (Webb, 1978). Where high energy prey items are available and resources are not limited, individual growth is likely greatest. Conversely, where resources are limited, or occur in pulses, both inter and intraspecific interactions for given resources, as a function of species densities or abundance, may also influence an individual's growth (Bystrom & Andersson, 2005; Claessen, de Roos, & Persson, 2000; Goldberg & Novoplansky, 1997; Heermann, Scharf, van der Velde, & Borcherding, 2014).

Within size‐structured populations, the effects of these interactions on individuals can shift dramatically based on the size of the individual and the resources that are available (Chase et al., 2002; Claessen et al., 2000; Polis, Myers, & Holt, 1989). Larger size may enable an individual to take advantage of an additional food subsidy, especially where morphometric constraints such as gape size can limit an individual's ability to access certain resources (e.g., piscivory), which may confer a growth advantage over smaller individuals (Armstrong et al., 2013; Juanes, Buckel, & Scharf, 2002; Persson, Bystrom, & Wahlstrom, 2000). Yet, where these alternative prey options or scenarios do not exist, larger individuals may actually be at a disadvantage due to disproportionate increases in metabolic costs with increased size (Brown, Gillooly, Allen, Savage, & West, 2004; Claessen et al., 2000).

Anadromous juvenile salmonids make relatively rapid transitions between freshwater, estuarine, and marine environments, resulting in large pulses of individuals entering or transiting habitats over a short period. These ontogenetic shifts in habitat use can lead to rapid increases in localized fish density, where inter and intraspecific interactions can potentially limit growth or survival to the next life stage and/or environment as the timing and magnitude of food availability and conspecific abundance changes. These interactions may be particularly important in juvenile Chinook salmon (*Oncorhynchus tshawytscha*) due to their migration timing and prolonged residence in these habitats (Healey, 1991; Healey & Groot, 1987). Within Puget Sound, Chinook salmon typically rear in the nearshore marine waters from June to September before migrating to the ocean to mature (Rice et al., 2011). Relatively high abundance of several small pelagic forage fish species is also present during this period, most notably Pacific herring (*Clupea pallasii*), although local abundances are known to vary considerably (Reum, Essington, Greene, Rice, & Fresh, 2011; Rice, Duda, Greene, & Karr, 2012). Juvenile Chinook salmon feed primarily on zooplankton and terrestrial insects, while residing in nearshore marine waters (Beauchamp & Duffy, 2011; Duffy, Beauchamp, Sweeting, Beamish, & Brennan, 2010; Kemp, Beauchamp, Sweeting, & Cooper, 2013; Osgood et al., 2016). Ontogenetic shifts in diet composition of juvenile salmonids are common, including for Chinook salmon, and are ultimately related to individual size (Brodeur, Francis, & Pearcy, 1992; Daly, Brodeur, & Weitkamp, 2009). As larger juvenile salmonids shift toward piscivory, Pacific herring have long been known as a prey resource in Puget Sound and along the west coast of the United States (Duffy et al., 2010; Emmett, Miller, & Blahm, 1986; Healey, 1980). While these associations have been well documented, the relative importance of piscivory on individual growth, the effect on the relationship between size and growth, and how it varies with species abundance and distributions are poorly understood.

Our goal was to evaluate the spatial and temporal variation in individual Chinook salmon growth rates and changes in the relationship between size and growth as a function of both Chinook salmon and herring abundance as well as individual size. Specifically, we asked, (1) where and when do Chinook salmon and herring abundance and size differ; (2) how do individual growth rates vary in space and time; (3) what is the spatial and temporal relationship between individual size and growth rate; and (4) how does the abundance and size of Chinook salmon and herring effect the observed relationship between size and growth.

## 2 METHODS

### 2.1 Study area

**Figure 1:**
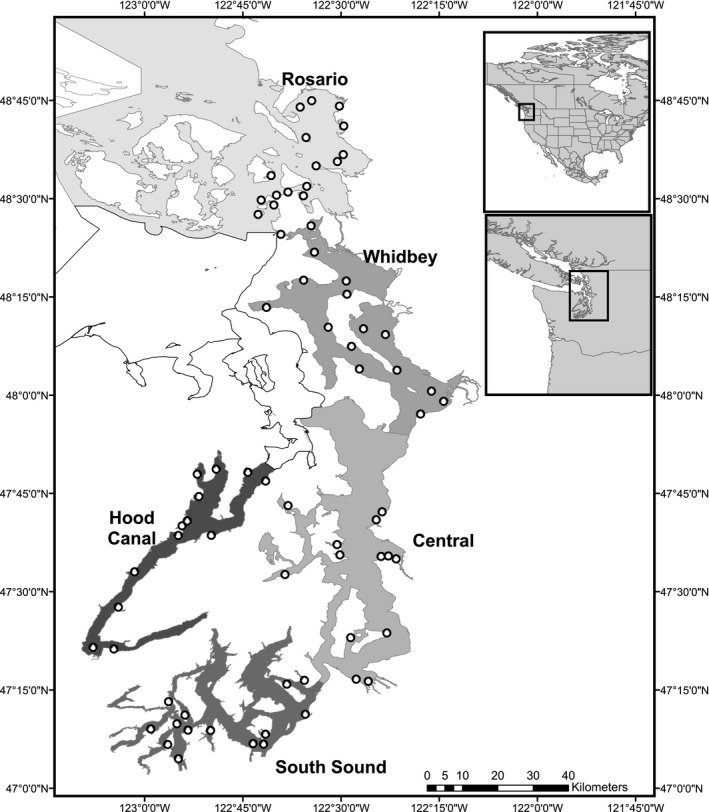
Map of Puget Sound, WA with sampling locations (open circles) and sub‐basin designations (shaded areas)

**Figure 2:**
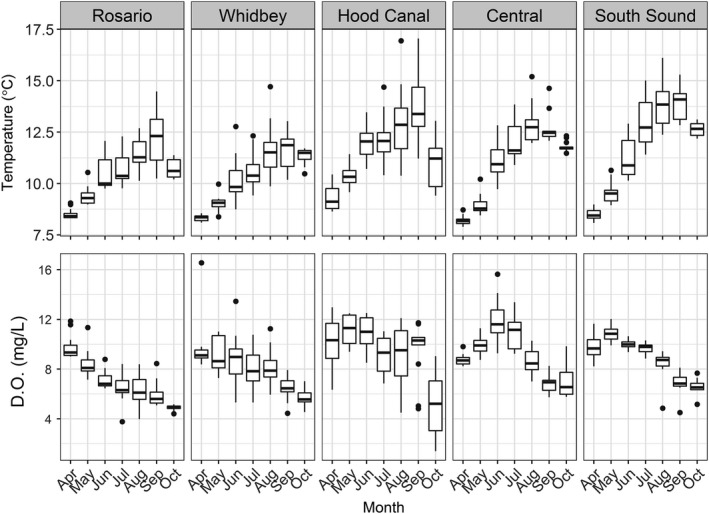
Mean surface (integrated top 6 m) temperature (°C) and dissolved oxygen (mg/L) measurements by month and sub‐basin. Boxes represent 25% to 75% quantiles, horizontal lines are medians, and vertical lines represent values within 95% CI

Puget Sound is a partially mixed fjord estuary complex driven by large and seasonal freshwater inputs, and significant ocean exchange through the Strait of Juan de Fuca and Admiralty Inlet (Figure 1). Tides are mixed, semidiurnal series with magnitudes ranging between 2.6 and 4.4 m (Mofjeld & Larsen, 1984). A series of sills and benches, along with variable inputs from large rivers, result in significant stratification and broadly variable residence times (0.7–73 days; Ebbesmeyer, Word, & Barnes, 1988; Babson, Kawase, & MacCready, 2006). Integrated mean surface (6 m) temperature and dissolve oxygen values vary seasonally, and differences among sub‐basins are relatively inconsistent (Figure 2). Puget Sound has historically supported relatively large populations of Chinook salmon and Pacific herring, although both species have declined to varying degrees in different basins of Puget Sound (Ford, 2011; Greene, Kuehne, Rice, Fresh, & Penttila, 2015; Rice et al., 2011). For this study, Puget Sound was stratified into five major sub‐basins (Rosario, Whidbey, Central, South, and Hood Canal) based upon oceanography and freshwater input. Sites were distributed within each sub‐basin to represent major habitat types (e.g., large embayments, small embayments, river deltas, and exposed shorelines; Figure 1.). Depths of sites varied within and among sub‐basins and ranged between 5 and 60 m.

### 2.2 Fish sampling

Sampling occurred at all sites once a month between April and October 2011. Fish were collected using a modified surface trawl (10 m W × 3 m D) with 6 mm mesh in the cod end, towed between two vessels via 50 m tow lines (Rice et al., 2012). Trawls were made against the current (when present) at approximately two knots through the water for a duration of 10 min. The mean volume of water sampled per tow was 15,434 m^3^. A subset of tows (*n* = 24 of 714) were shortened to 5 min when large quantities of gelatinous zooplankton were encountered and a longer duration was not possible (Greene et al., 2015). We calculated the number of individuals captured per minute for these tows then expanded to represent a 10 min tow before analysis. At the conclusion of each tow, all fish were brought on board and sorted into live holding tanks with flow‐through sea water from each site. All fish were identified to species and counted. Up to 25 individuals of each species were measured to fork length where possible, or total length for species without a forked caudal fin. To identify natural origin individuals from hatchery reared fish, all salmon were checked for external marks and checked for coded‐wire tags (CWT) using a CWT wand.

### 2.3 Sample processing

Individual growth rates were assessed by evaluating concentration of insulin‐like growth factor‐1 (IGF‐1) in individual Chinook salmon captured throughout Puget Sound. Insulin‐like growth factor‐1 (IGF‐1) is a plasma hormone known to stimulate and support cellular growth in individual animals (Mommsen, 1998). Several factors may affect the production of IGF‐1 including photoperiod, temperature, and nutrition (e.g., food quality and quantity; Picha, Turano, Beckman, & Borski, 2008; Beckman, 2011). More recently, researchers have used IGF‐1 levels to compare growth rates in fishes, and in particular salmonids, across a variety of conditions (e.g., temporal/seasonal, physical etc. (Beckman, Larsen, Lee‐Pawlak, & Dickhoff, 1998; Beckman, Fairgrieve, Cooper, Mahnken, & Beamish, 2004; Larsen, Beckman, & Cooper, 2010; Stefansson et al., 2012) and as a function of individual size (Beaudreau, Andrews, Larsen, Young, & Beckman, 2011).

Due to the limitations of comparing IGF‐1 concentrations across seasons (Beaudreau et al., 2011; Beckman, 2011), we limited our analyzes to fish sampled during the summer months (June‐August).A subset of up to six individuals of both marked (hatchery origin) and unmarked (presumed natural origin) Chinook salmon from each site were killed for growth analysis. Each fish was measured and weighed, and a blood sample was taken immediately after the fish was killed. Blood was drawn using heparinized Nielsen tubes and placed into 5‐ml micro tubes and stored on wet ice for no more than 2 hr before being spun in a centrifuge for 5 min at 3000 g. Plasma was then separated from the red blood cells and immediately frozen. Samples were transferred to the −80°C freezer within 12 hr and stored until laboratory processing occurred.

In brief, IGF‐1 was measured in plasma using a fluorescence‐based immunoassay following the methods of Ferriss, Trudel, and Beckman (2014). All samples were processed and analyzed in duplicate to assess coefficient of variation (CV). Samples (*n* = 63) that had a CV that exceed 10% were excluded from the study.

### 2.4 Statistical analysis

Salmon and herring abundances were summarized by month and sub‐basin using total biomass catch per unit effort (CPUE, g/tow). Individual size (fork length) of Chinook salmon and Pacific herring as well as mean IGF‐1 concentrations for individual Chinook salmon were compared among sub‐basins, months, and origin (hatchery vs. presumed wild) using linear regression techniques. All single factors and first‐order interactions were compared in our analysis. Prior to analysis, all values outside the 95% CI were removed from the dataset to reduce the effect on the overall mean within each of the groups. A total of two samples were removed from the entire dataset.

Mixed effects multilevel regression models were used to evaluate how IGF‐1 concentrations and size of individual Chinook salmon were related throughout our study area and how Chinook salmon and herring abundance as well as relative size influenced the observed variability. Multilevel regression models are useful for datasets with inherent grouping structure (Gelman & Hill, 2007). Groups for this particular analysis were based on observed patterns and previous work highlighting differences in Chinook salmon and herring size and abundance among the sub‐basins and the temporal shifts in these observed patterns from month to month. (Reum et al., 2011; Rice et al., 2011, 2012). Within our framework, each sub‐basin × month (*n* = 15) was treated as a random effect; whereby, we estimated individual slopes and intercepts for each parameter within each month × sub‐basin group. Estimating unique slopes and intercepts for each group allowed us account for potential differences in mean IGF‐1 concentrations and evaluate potential variability in the relationship between size and growth (i.e., slope) among groups. Preliminary analysis indicated our response (IGF‐1 concentration) was normally distributed; therefore, all models were evaluated using a Gaussian error structure, and all predictors/covariates were transformed accordingly (Table 1). Models took the form:

$$y_{i}∼N\;\left(\alpha_{j[i]}\;+\;\beta_{j[i]}x_{i},\sigma_{i}^{2}\right),\;\;\text{for}\;i\;=\;1,\ldots ,\;n$$

$$\begin{array}{cc} \left(\begin{array}{c} \alpha_{i}\\ \beta_{i} \end{array}\right) & ∼N\left(\left(\begin{array}{c} \gamma_{0}^{\alpha }+\gamma_{1}^{\alpha }u_{1j}\ldots +\gamma_{k}^{\alpha }u_{kj}\\ \gamma_{0}^{\beta }+\gamma_{1}^{\beta }u_{1j}\ldots +\gamma_{k}^{\beta }u_{kj} \end{array}\right),\left(\begin{array}{cc} \sigma_{\alpha }^{2} & \rho \sigma_{\alpha }\sigma_{\beta }\\ \rho \sigma_{\alpha }\sigma_{\beta } & \sigma_{\beta }^{2} \end{array}\right)\right),\;\\ & \text{for}\;k\;=\;1,\ldots ,K\;\text{predictors and}\;j\;=\;1,\ldots ,J\;\text{groups}, \end{array}$$

where $\gamma_{0}^{\alpha }$ is the intercept, $\gamma_{0}^{\beta }$ the slope for individual fork length, and the $\gamma_{k}^{\alpha }$'s and $\gamma_{k}^{\beta }$'s represent the intercepts and slopes, respectively, for each group (*k*) and predictor (*j*). Finally, the terms, $\sigma_{\alpha }^{2}$ and $\sigma_{\beta }^{2}$ are the standard deviations for the group level intercept and slope, respectively, while ρ is the between‐group correlation parameter. Because the primary interest was to evaluate the relationship between individual size and growth at each level of our group effects, we chose to allow for correlation between random slope and intercept estimates and estimated the covariance as appropriate.

**Table 1:** Model terms, transformations, number of parameters, and the level at which the term is measured for first stage model selection procedure

Abbr	Description	Transformation	Level	*n*
Length	Individual fork length	Standardized*by month of capture	Individual	638
HA:CA	Ratio of herring and Chinook salmon abundance	*x* ^1/4^	Group	15
Mean 6 m D.O	Mean dissolved oxygen, surface 6 m integrated	None	Group	15
Mean 6 m Temp	Mean temperature, surface 6 m integrated	None	Group	15

Our initial set of predictors included a separate term for Chinook salmon abundance, Pacific herring abundance, the ratio of the species abundances for each group, and an individual origin (hatchery vs. natural production) term. However, exploratory analysis revealed significant correlation among the individual abundance metrics and the ratio term. Given the high degree of correlation among terms, we removed the individual abundance terms and used only the ratio term for our modeling exercise. Similarly, individual origin showed no effect of origin on growth (*F *=* *1.5, *p *=* *.223), and therefore, both hatchery and naturally produced individuals were pooled for subsequent analyzes. We also included mean temperature and dissolved oxygen measurements integrated over the surface 6 m to represent environmental variability among basins through time. Candidate models for our selection procedure included all individual terms as well as the potential interaction between individual size and the abundance ratio term (Table 1).

Because one of our primary goals was to assess the relationship between size and growth within and among our groups, all models were forced to include the term for individual fork length. To account for noise within our models due to potential differences in individual size of Chinook among groups, and to aid interpretation of model results, we standardized fork lengths (FL) to a mean of zero within each month × sub‐basin group using the following equation:

(1) $${\text{FL}}_{s}\;=\;\frac{{\text{FL}}_{i}-\mu_{j}}{\sigma_{j}}$$

where, standardized fork length, FL_*s*_, is equal to the difference between and individual fork length (mm), FL_*i*_ and the mean fork length, μ_*j*_, for month *j*, divided by the standard deviation of fork length in month *j*, σ_j_.

We used a second‐stage procedure to evaluate how size‐structured abundance of Pacific herring influenced IGF‐1 concentrations. The second‐stage procedure built upon the best model from the initial selection process and included a parameter, *h*_*x*_ *,* that represented the proportion of total herring abundance that fell into a given size category relative to each individual Chinook salmon.

as follows:

(2) $$h_{x}\;=\;\frac{n_{ij}}{N_{j}}*a_{j}\;\text{where},$$

*n*_*ij*_ is the number of individual herring of length ≤*x*% of the length of the *i*th individual, *N*_*j*_ is the total number of herring measured in the *j*th group, and *a*_*j*_ is the abundance of herring for the *j*th group. We evaluated these proportions at 10% intervals for *x* = 30%–80% which allowed for exploration of potential size thresholds that influenced IGF‐1 concentrations in individual fish. Interval ranges were based on values in the published literature and inspection of our data (Beauchamp & Duffy, 2011). All interval terms, as proportions, were logit transformed prior to inclusion in the model (Warton & Hui, 2011).

Models were initially fit using maximum likelihood methods to allow for comparison of candidate models with different sets of fixed effects using AICc (Anderson & Burnham, 2002; Burnham & Anderson, 2002; Faraway, 2006; Zuur, Ieno, Walker, Saveliev, & Smith, 2009). Models with AICc < 2 were considered indistinguishable, and model weights (Akaike weights; Burnham & Anderson 2002) were calculated to determine the best fit model. Best fit models were then re‐estimated using restricted maximum likelihood (REML) to obtain more precise coefficients estimates for the selected parameters (Faraway, 2006; Zuur et al., 2009). Explained variance (*R* ^2^), both conditional and marginal, was estimated following methods developed specifically for use with mixed models (Johnson, 2014; Nakagawa & Schielzeth, 2013). Lastly, we estimated variable importance by summing the model weights over all candidate models that included each explanatory term. All analysis was performed using R statistical software (R Core Team 2015; version 3.2.3). Models were fit and evaluated, and model selection procedures were run using the lme4 (Bates, Mächler, Bolker, & Walker, 2015) and MuMln (Barton, 2016) packages.

## 3 RESULTS

### 3.1 Abundance and size

**Figure 3:**
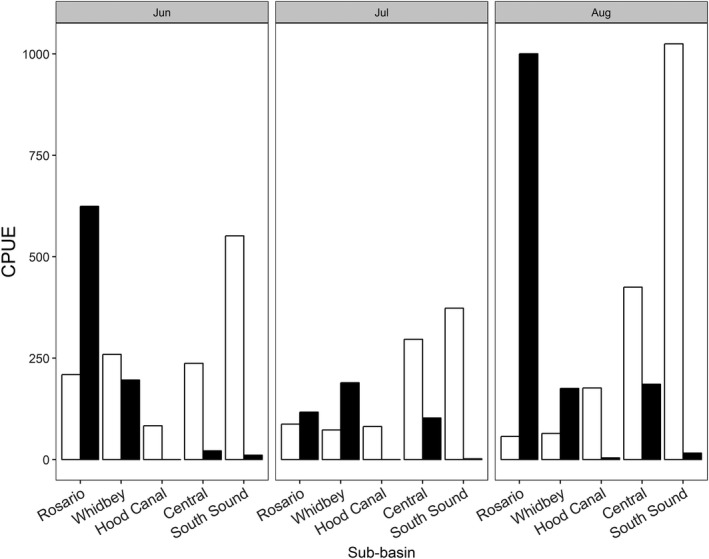
Catch per unit effort (g/tow) for Chinook salmon (unfilled) and Pacific herring (filled.) by sub‐basin and month

Chinook salmon CPUE varied spatially within and among sub‐basins and seasonally among months, both within and among sub‐basins (Figure 3). Mean CPUE was highest in the South Sound (649.9 g) and lowest in Hood Canal (113.7 g). Chinook salmon CPUE was highest in South Sound during all months. Peak CPUE for Chinook salmon biomass in the northern sub‐basins (Rosario and Whidbey) occurred in June and generally decreased through August, whereas CPUE generally increased from June through August in the Central and South Sound sub‐basins. CPUE in Hood Canal was relatively low in June and July before peaking in August.

Pacific herring CPUE also varied spatially and seasonally among and within sub‐basins (Figure 3) with a clear latitudinal gradient in herring CPUE among the sub‐basins. Mean CPUE of Pacific herring was lowest in Hood Canal and South Sound (1.7 and 9.7 g, respectively) and highest in the Rosario sub‐basin (580.5 g). Pacific herring were captured in all months in all sub‐basins with the exception of Hood Canal. Herring CPUE was generally highest in August in all sub‐basins except Whidbey, while months with the lowest CPUE were inconsistent among sub‐basins. The Rosario sub‐basin had the highest herring CPUE by an order of magnitude in all months except for July.

**Figure 4:**
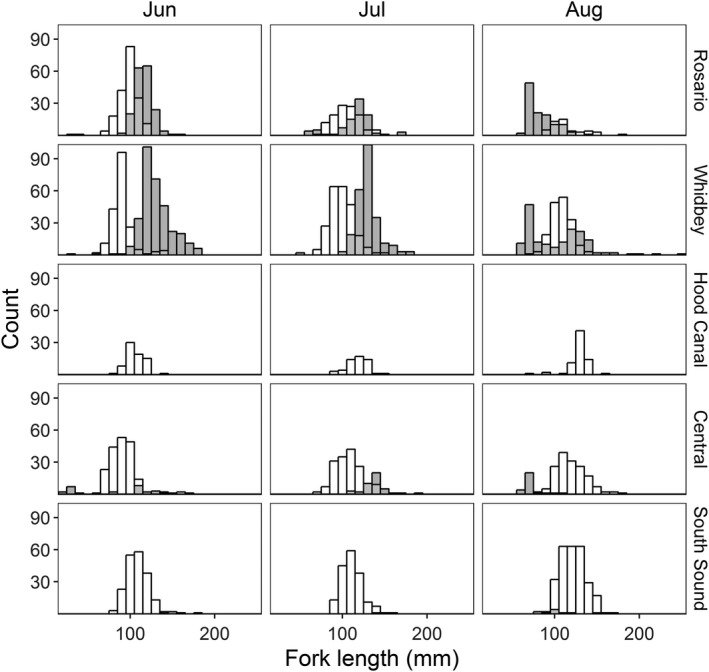
Length frequency histograms of individual fork length (mm) for Pacific herring (filled) and Chinook salmon (unfilled) by sub‐basin during June, July, and August

Individual size of Chinook salmon varied by month (*F* = 134.9, *p* < .001; Figure 4) and to a lesser degree among sub‐basins (*F* = 22.9, *p* < .001; Figure 4). In general, the mean size of Chinook salmon in all sub‐basins increased through time with the exception of both Hood Canal and Whidbey sub‐basins, where a small number of larger fish were captured early in the year resulting in an increased mean size during those months. Differences among sub‐basins were largely due to increased variability, or broader range of sizes, in the Whidbey and Central sub‐basins.

Individual herring size (FL) also varied by month (*F* = 275.2, *p* < .001) and among sub‐basins (*F* = 58.9, *p* < .001; Figure 4). Monthly changes in size were much greater than differences among sub‐basins. Unlike Chinook, herring in all regions were largest in June before showing a consistent decline in mean size through August. From June through the end of the sampling period, Pacific herring in South Sound were, on average, larger than in any other sub‐basin.

### 3.2 Model selection

A total of 638 Chinook salmon were included in analysis of IGF‐1 concentration during June‐August 2011 and represented all five sub‐basins (Table 2). Twelve candidate models were evaluated to explain variability in IGF‐1 concentrations for Chinook salmon among and within our sub‐basin × month groups. Our selection criteria (ΔAICc < 2; Anderson & Burnham, 2002) suggested the top four models were plausible fits yet model weights indicated the top two models outperformed all others (0.34/0.13 = 2.6; Table 3). The top two models only differed by the inclusion of temperature and the model with this term did not add considerable explanatory power (Δ*R* ^2^ = .003). In contrast, the abundance ratio term and the interaction between individual size and the abundance ratio term were included in each of the top four models. Models that included term(s) for abundance ratios performed better than a model with only individual size (Table 3). Variable importance metrics indicated the abundance ratio term as well as the interaction between abundance ratios and size were most important (0.94 and 0.91, respectively), while the temperature (0.50) and dissolved oxygen (0.27) terms were less important.

**Table 2:** Number of Chinook salmon sampled for IGF‐1 by sub‐basin basin and month

Sub‐basin	June	July	August	Total
Rosario	49	53	31	133
Whidbey	37	46	35	118
Hood Canal	28	24	37	89
Central	49	38	47	134
South Sound	50	50	64	164
Total	213	211	214	638

**Table 3:** Model selection results for first stage selection procedure, including terms in each model, the number of parameters (K), log likelihood estimated using Maximum likelihood (logLik), AICc values (AICc) and differences (ΔAICc), and model weights

Model	*K*	logLik	AICc	ΔAICc	Weight
Length + HA:CA + Mean 6 m Temp + Length*HA:CA	9	−2541.26	5113.88	0	0.347
Length + HA:CA + Length*HA:CA	8	−2543.61	5114.01	0.132	0.324
Length + HA:CA + Mean 6 m DO + Length*HA:CA	9	−2542.45	5115.86	1.974	0.129
Length + HA:CA + Mean 6 m DO + Mean 6 m Temp + Length*HA:CA	10	−2540	5116.1	2.219	0.114
*Length*	6	−2551.33	5119.3	5.414	0.023
*Length + Mean 6 m Temp*	7	−2549.42	5119.76	5.883	0.018
*Length + Mean 6 m DO*	7	−2550.6	5120.59	6.712	0.012
*Length + HA:CA + Mean 6 m Temp*	8	−2547.06	5121.17	7.292	0.009
*Length + HA:CA*	7	−2549.36	5121.26	7.375	0.009
*Length +Mean 6 m DO + Mean 6 m Temp*	8	−2548.87	5121.59	7.71	0.007
*Length + HA:CA + Mean 6 m DO*	8	−2548.12	5122.78	8.894	0.004
*Length + HA:CA + Mean 6 m DO + Mean 6 m Temp*	9	−2545.9	5123.53	9.652	0.003

### 3.3 IGF‐1 concentrations

**Figure 5:**
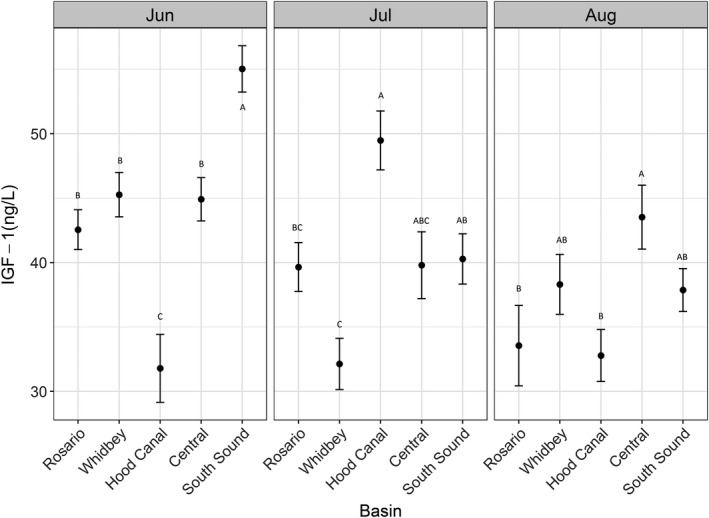
Mean IGF‐1 concentration (±*SE*) for Chinook salmon by sub‐sub‐basin and month. Letters denote sub‐basins within a month with significantly different means (Tukey HSD post hoc test)

Mean IGF‐1 levels varied significantly by month (*F* = 18.0, *p* < .001) as well as among sub‐basins (*F* = 6.2, *p* < .001; Figure 5). In addition, the estimated standard deviation of group intercepts suggested considerable between‐group differences in mean IGF‐1 concentrations (Table 4). Overall, mean IGF‐1 concentrations were highest in Central and South Sound and lowest in Hood Canal. In general, mean concentrations declined consistently through time in all sub‐basins with the exception of Hood Canal where concentrations remained low in both August and June but showed a peak in July. While concentrations differed among sub‐basins and months, significant differences in mean IGF‐1 concentrations between months did not occur in all sub‐basins (Figure 5). Chinook captured in the Central and Hood Canal sub‐basins had relatively consistent mean IGF‐1 concentrations between June and August, whereas mean values in the remaining sub‐basins decreased considerably. Fish from Hood Canal had the lowest mean IGF‐1 concentrations in both months among all sub‐basins. In general, IGF‐1 values were more variable in August than in June for all sub‐basins except Hood Canal, which had the highest variability in IGF‐1 concentrations in June. Whidbey sub‐basin had the least variable IGF‐1 values in both June and August.

**Table 4:** Estimates and standard errors for fixed effects and standard deviations and correlation for group effects from best fit model. Coefficient estimates obtained using REML


Fixed effects	Est	*SE*
int	40.992	2.672
HA:CA	0.788	2.855
Length	−0.02	1.483
HA:CA*length	5.191	1.518


Group effects	*SD*	Corr
Sub‐basin × month	5.479	
Length	2.144	0.47
Residual	12.783	

### 3.4 Relationship between individual size and IGF‐1 concentrations

**Figure 6:**
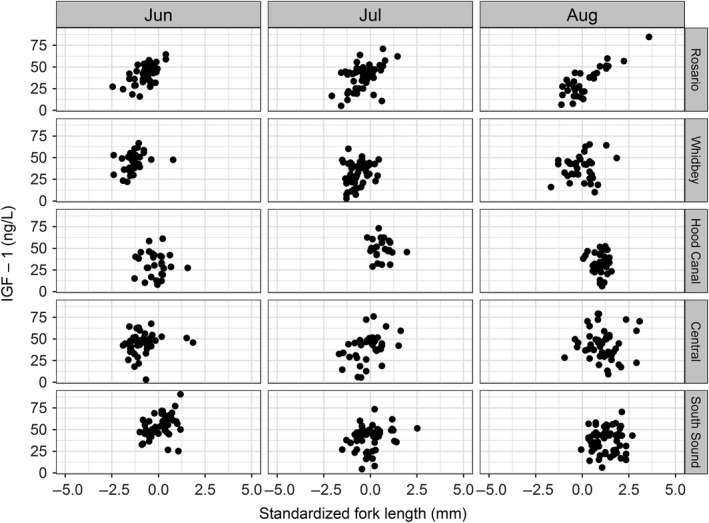
Individual Chinook salmon IGF‐1 values as a function of fork length by sub‐basin and month

The estimated standard deviation for group slopes also suggested the relationship between size and growth varied among our sub‐basin × month groups, although differences were not as substantial as observed for mean IGF‐1 concentrations (Table 4). In general, the relationship between size and growth was positive, although the pattern was not ubiquitous or consistent among groups (Figure 6, Table 5). Slopes were generally more variable among sub‐basins than among months, while the strength of the relationship declined from north to south. Size and growth were most strongly correlated in Rosario sub‐basin in all months. The relationship between size and IGF‐1 concentration in Hood Canal was unique in that it showed an apparent negative relationship across months with some variability.

**Table 5:** Group (random) errors and regression coefficients (slope and intercept) from the best fit model (see Table 3). Regression coefficients integrated the fixed effects, group errors, and group specific values for the abundance ratio term

Group	Group errors		Coefficients	
	Intercept	Slope	Intercept	Slope
CentralAug	0.697	−1.970	42.330	2.230
CentralJul	−1.941	0.890	39.660	4.850
CentralJun	3.976	−0.080	45.400	2.750
Hood CanalAug	−7.551	−1.771	33.750	0.260
Hood CanalJul	5.950	1.219	46.940	1.200
Hood CanalJun	−7.094	−1.492	33.900	−1.510
RosarioAug	−0.755	0.923	41.850	11.530
RosarioJul	−0.314	0.640	41.530	6.200
RosarioJun	1.538	0.975	43.570	7.770
South SoundAug	−3.609	−0.860	37.660	0.960
South SoundJul	−1.214	0.839	39.990	2.240
South SoundJun	8.400	3.209	49.690	5.130
WhidbeyAug	0.616	−1.714	42.620	4.930
WhidbeyJul	−5.783	−1.087	36.210	5.480
WhidbeyJun	7.084	0.278	48.810	5.100

### 3.5 Influence of Chinook salmon and Pacific herring abundance

**Figure 7:**
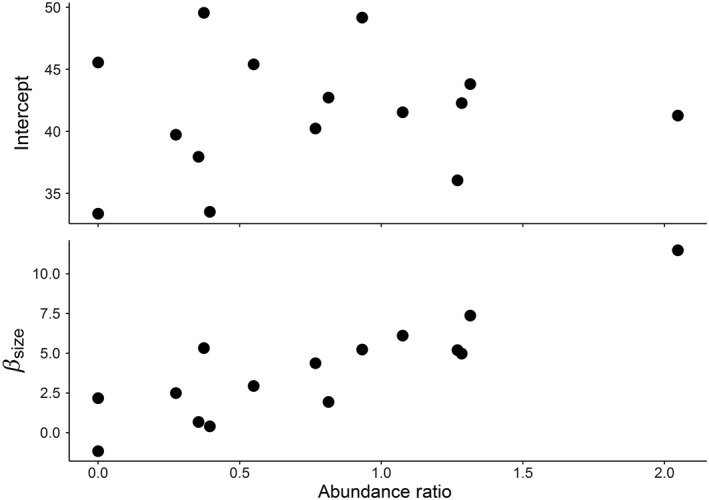
Scatterplots of (a) mean IGF‐1 concentrations and (b) the slope of size to growth (β_size_) as a function of abundance ratio. A ratio of 1.0 reflects equal abundance of Chinook salmon and Pacific herring. Ratios above 1.0 indicate Pacific herring abundance higher than Chinook salmon abundance. Slopes include the effect of the interaction between individual size and the ratio of Pacific herring and Chinook salmon abundance

**Figure 8:**
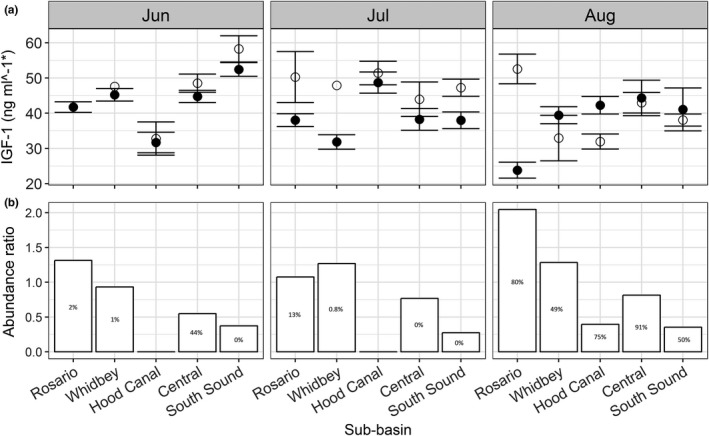
(a) Mean growth rates for small (<120 mm; filled circles) and large (>120 mm; open circles) individual Chinook salmon. Break point determined by calculating the size at which the smallest observed herring length (48 mm) represented 40% of a given Chinook salmon length. (b) Abundance ratio values by sub‐basin and month. Values above 1.0 indicate higher relative proportions of Pacific herring. Percentages represent highest possible proportion of Pacific herring that fall into the 40% threshold using the smallest observed Pacific herring and largest observed Chinook salmon

Our best fit model included the abundance ratio term as well as an interaction between abundance ratio and individual size. Model fits of the abundance ratio term suggested a mildly positive effect on IGF‐1 concentrations if the abundance ratio was skewed toward Pacific herring (Figure 7a). However, the estimate for the interaction term between individual size × abundance ratio was clearly positive and had a strong positive effect on the relationship between IGF‐1 concentration and individual size. The slope of the relationship between size and IGF‐1 concentrations increased linearly where Pacific herring abundance was greater than Chinook abundance (Figure 7b). IGF‐1 concentrations were also elevated for average and above average size individual Chinook salmon when Pacific herring were more abundant than Chinook salmon (Figure 8a).

The ΔAICc values and model weights for our second‐stage modeling exercise suggested the best model included the term representing the presence and abundance of herring that were less than or equal to 40% of an individual Chinook salmon's length (Table 6). Where the proportion of the Pacific herring population that fell below this threshold was the greatest, above average size Chinook salmon had elevated IGF‐1 concentrations (Figure 8b). The model coefficient (2.378, *SE* = 1.571) also indicated a positive effect on mean IGF‐1 concentration where greater abundances of herring at less than or equal to 40% of an individual Chinook salmon occurred. However, the term added relatively little explanatory power to the overall model (Δ*R*_marg_ = 0.013).

**Table 6:** Model selection results from second‐stage selection procedure to evaluate the influence of size‐structured Pacific herring abundance relative to each individual Chinook. Best fit model in bold. Includes coefficient estimate for the added term (each term was added to best fit model from initial selection procedure), log likelihood (logLik), AICc values (AICc) and differences (ΔAICc), and model weights

	Estimate	logLik	AICc	ΔAICc	Weight
Herring 30%	0.095	−2542.92	5104.133	3.633	0.084
**Herring 40%**	**2.378**	**−2541.1**	**5100.5**	**0.000**	**0.518**
Herring 50%	−0.657	−2542.72	5103.734	3.234	0.103
Herring 60%	−0.160	−2542.91	5104.115	3.615	0.085
Herring 70%	−0.684	−2542.66	5103.615	3.115	0.109
Herring 80%	−0.683	−2542.75	5103.785	3.285	0.100

## 4 DISCUSSION

IGF‐1 concentrations and the relationship between size and IGF‐1 concentration of individual Chinook salmon were influenced by the co‐occurrence of Pacific herring. This study provides the first spatially explicit evaluation of variability in Chinook salmon growth throughout the nearshore waters of greater Puget Sound and present a plausible mechanism for the observed spatial and temporal differences. Correlations between size and IGF‐1 concentrations were generally positive and strongest where herring were in higher abundance than Chinook salmon. Where and when small herring were most abundant, average and above average sized Chinook had higher IGF‐1 concentrations than smaller individuals did within a given basin and month (Figure 8). In contrast, where herring abundance was low and Chinook salmon abundance high, the relationship between size and IGF‐1 concentration was rather weak and included a high degree of variation (Figure 7b). We propose the observed variability in the size‐growth relationship reflects differences in community structure and a localized, size‐mediated switch to piscivory, both of which ultimately influence growth opportunity for individual Chinook salmon.

### 4.1 Growth influenced by variation in species abundance

We observed significant spatial variation and temporal differences for Chinook salmon and Pacific herring abundance. These results largely corroborated previous studies evaluating recent trends in small pelagic fish abundance and distribution in Puget Sound (Reum et al., 2011; Rice et al., 2011, 2012). Current fish assemblages in northern Puget Sound are known to be more diverse and have higher abundances of pelagic species than areas within the main sub‐basin (Rice et al., 2012), but the impact of these spatial and temporal differences in community composition on the dynamics of local fish populations, including salmon, has not yet been documented.

Shifts in distribution or changes in local abundance or density can affect or alter both inter and intraspecific interactions and subsequently impact individual growth (Husebø, Slotte, & Stenevik, 2007; Jansen & Burns, 2015; Jenkins, Diehl, Kratz, & Cooper, 1999; Lorenzen & Enberg, 2002). Total and relative herring abundance appears to influence individual growth of Chinook salmon (as assessed via IGF‐1 concentrations). Where Pacific herring were more abundant than Chinook salmon, the effect on mean growth was slightly negative across the entire length range of Chinook salmon in our study. Diet overlap among Pacific herring and Chinook salmon along the west coast of North America as well as within, and adjacent to, the epi‐pelagic habitats of Puget Sound is considerable and relatively consistent among years (Hill, Daly, & Brodeur, 2015; Kemp, 2014; Osgood et al., 2016). Bioenergetics modeling of Chinook salmon growth in nearshore marine habitats within Puget Sound has indicated clear sensitivity to consumption rates given the range of temperatures experienced during summer months (Beauchamp & Duffy, 2011). Given this sensitivity, the observed spatial variability in mean IGF‐1 concentration could simply reflect different feeding rates. But while differences in consumption rates may be influenced by the known spatial and temporal distribution of prey in Puget Sound (Cooney, 1971; Hebard, 1956), and the potential of resource limitation (J. Keister, unpublished data), it is plausible that the observed reduction in mean growth rates indicates increased competition where herring were more abundant. These results follow conventional theory found in much of the literature concerning effects of competition on growth (Gurevitch, Morrison, & Hedges, 2000). While such competitive interactions between Chinook salmon and herring have been proposed, they have not been linked to growth consequences for individual salmon (Beauchamp & Duffy, 2011; Kemp, 2014).

Our dataset did not allow for direct assessment of competition between Pacific herring and Chinook salmon, yet our results may allow us to hypothesize about the potential for such interactions. Specifically, the negative impact on IGF‐1 concentration, where Pacific herring were more abundant and/or the weakened relationship between size and IGF‐1 concentrations where Pacific herring were relatively large and/or more abundant than Chinook salmon, is the plausible result of inter or intraspecific competitive interactions, respectively. So while increased herring abundance likely increases competitive interactions, the presence of herring and in particular small herring, may provide refuge from adverse impacts on growth for above average Chinook salmon individuals.

### 4.2 Increased growth opportunity due to size‐mediated shift to predation

Although we observed a negative correlation between herring abundance and mean growth of Chinook salmon, we observed a strong positive interaction between the abundance ratios and individual size that influenced how growth and size were related within groups. Where herring were most abundant (Rosario and Whidbey sub‐basins), the relationship between size and growth was clearly positive. Within such groups, mean growth rates were higher for average and above average sized Chinook salmon (Figure 8a). Thus, although overall mean growth rates were lower for these groups, it was likely due to the poor growth rates observed for small individuals and not the favorable growth rates for larger fish. In contrast, where herring were less abundant or absent, the relationship between size and growth was either weak or nonexistent, and growth rates were similar for large and small individuals (Figures 7 and 8). We may expect similar growth rates between fish of different sizes where access to prey resources is not morphometrically limited by size and where prey resources have considerable spatial variability or patchiness, in which case, larger size may not provide a benefit and may actually confer a metabolic disadvantage (Persson, Leonardsson, de Roos, Gyllenberg, & Christensen, 1998).

The observed potential growth benefit for average to above average individual Chinook salmon when and where herring were abundant may be indicative of predation on Pacific herring by Chinook salmon; where herring become prey rather than potential competitors. Size‐mediated predator–prey interactions are widely reported in the literature including in many fishes (Dorner, Hulsmann, Holker, Skov, & Wagner, 2007; Persson et al., 2004). Intraguild predation is unique among populations or species that experience significant overlap in diet composition and possible competition for food resources, where large individuals have a potential metabolic disadvantage, and where smaller conspecifics are present (Claessen et al., 2000; Gårdmark et al., 2015; Persson et al., 2000, 2004; Polis et al., 1989). Examples of intraguild predation dynamics are well documented for pelagic species throughout the world (Canales, Law, & Blanchard, 2015; Gårdmark et al., 2015). While we present no empirical evidence of Chinook salmon predation on herring from our study, herring have long been known as a prey resource for salmonids of different size and ages in Puget Sound and along the US west coast (Beauchamp & Duffy, 2011; Daly et al., 2009; Emmett et al., 1986).

We also evaluated the effect of the proportional abundance of herring within a given size class relative to each individual Chinook salmon as a measure of potential predation capability. While the predictor did not greatly improve the explained variation, our observed size threshold of 40% was similar to the threshold found in other size‐mediated predation studies of fish (Brodeur, Buchanan, & Emmett, 2014; Juanes, 2003; Juanes et al., 2002) as well as empirical data for Chinook salmon prey in Puget Sound (Beauchamp & Duffy, 2011). The greatest proportions of Pacific herring that fell under this threshold occurred in the northern sub‐basins where the relationship between size and growth was strongest (Figure 8b). The observed differences in sizes among sub‐basins may be indicative of variable spawn timing among herring stocks with Puget Sound. Puget Sound has several different stocks of Pacific herring and while some are genetically distinct, most are distinguished by spawning location and timing (Stout et al., 2001). In general, Puget Sound herring populations spawn in February and March with the exception of the Cherry Pt population, at the northern extent of Puget Sound, which spawn between April and June. Late spawning stocks would likely have a larger proportion of smaller individuals present during the same time period and provide a potential food subsidy for above average sized Chinook salmon in that particular area. In contrast, where early spawning populations or stocks were present, young of the year herring would emerge earlier in the year and thus be larger during the period they overlap with juvenile Chinook salmon in the nearshore marine habitats.

Although environmental parameters can have significant effects on individual growth and or species distributions (Brander, 1995; Brandt, Gerken, Hartman, & Demers, 2009; Essington & Paulsen, 2010; Jonsson & Jonsson, 2009), we did not find any evidence for strong environmental effects on growth in our data set. The temperature term was included in one of our top models and coefficient estimates (−2.250, *SE* = 1.552) indicated IGF‐1 concentrations would decrease where surface temperatures increased. Yet the inclusion of the term did not significantly improve fit or explanatory power and variable importance measures suggested it was not as important as the other terms in the model. Furthermore, temperature and dissolved oxygen patterns within Puget Sound reveal strong seasonal patterns but differences among sub‐basins are rather weak with the exception of dissolved oxygen levels in Hood Canal (Figure 2). While these metrics can have significant impacts on individual growth, or the interpretation of tools used to assess individual growth (e.g., bioenergetics modeling), the lack of variability precluded them from providing useful information regarding differences in IGF‐1 concentrations for individual Chinook salmon.

### 4.3 Implications

Our study provides a causal explanation of spatial variability in growth and its relationship to individual size as influenced by conspecific abundance. These findings suggest that low abundances of juvenile herring may have important consequences for salmon populations as they are forced to switch to less energy‐rich prey and remain at sizes resulting in enhanced competition with conspecifics (e.g., Pacific herring). Understanding the potential drivers and differences in fish community dynamics and their potential impact on focal populations could have significant stock recovery implications for Chinook salmon in the Puget Sound.

Linking conspecific population dynamics to declining trends for focal salmonid populations has been documented in many systems (Cooney et al., 2001; Kallio‐Nyberg, Jutila, Jokikokko, & Saloniemi, 2006; Kitchell et al., 2000; Mantyniemi et al., 2012; Thayer, Field, & Sydeman, 2014). Declines and fluctuations in abundance or recruitment of small pelagic fish communities have been implicated in observed decreases in survival and growth for salmonids across the Northern Atlantic and Eastern Pacific Oceans (Chaput & Benoit, 2012; Jonsson, Jonsson, & Albretsen, 2016; Thayer et al., 2014). And although the processes that drive these trends may vary (Beaugrand & Reid, 2003; Lindegren, Ostman, & Gardmark, 2011), their effect on the productivity and success of salmonid populations remains a concern.

Marine survival of Chinook salmon in Puget Sound has declined since the early 1980's and remained low for several decades. It is widely believed that individual growth during the early marine portion of the life history increases the probability of juvenile salmon survival to subsequent life stages, and may determine overall marine survival of salmon populations in the Pacific Northwest (Beamish & Mahnken, 2001; Duffy & Beauchamp, 2011). Evidence suggests that faster growth and/or larger individual size during, and at the conclusion of, this early marine period leads to increased survival (Beamish, Mahnken, & Neville, 2004; Cross, Beauchamp, Moss, & Myers, 2009; Duffy & Beauchamp, 2011; Holtby, Andersen, & Kadowaki, 1990). Our results indicate a potential growth benefit for average to above average fish in sub‐basins where higher abundances of small herring provide an additional energy‐rich food subsidy. However, throughout Puget Sound, several herring populations or spawning stocks are known to be depressed or fluctuate far below historic abundance levels (Greene et al., 2015; Siple & Francis, 2016; Stout et al., 2001). Should the presence of herring indeed provide a greater growth opportunity to juvenile salmon, the observed trends in herring abundance could impact individual growth and thus overall survival.

Finally, while our results suggest such a relationship between herring presence and Chinook, there remains considerable variability in growth that cannot be explained by the selected predictors. Our study presents evidence of inter and intraspecific effects on individual growth of Chinook salmon in Puget Sound; however, we cannot disregard other potential factors not evaluated within our current framework. The Puget Sound food web is complex and undoubtedly affected by a number of potential bottom‐up and/or top‐down processes or interactions, any of which could impact individual growth (Busch, Harvey, & McElhany, 2013; Harvey, Williams, & Levin, 2012). Therefore, we must acknowledge the presence of such factors (i.e., environmental, productivity, human induced contaminants/urbanization etc.) and their potential role in driving the conditions observed in our dataset. In addition, while we may assume a causal relationship between size and growth and the interaction with herring abundance and size, future research that couples observed growth with diet composition and/or stable isotope analysis may be necessary to provide an empirical mechanistic link to support the conclusion.

## CONFLICT OF INTEREST

None declared.
